# Frendak to Phenis to Breivik: An Examination of the Imposed Insanity Defense

**DOI:** 10.3389/fpsyt.2014.00172

**Published:** 2014-12-01

**Authors:** William Donald Richie, Farzana Alam, Lalitha Gazula, Harold Embrack, Milankumar Nathani, Rahn Kennedy Bailey

**Affiliations:** ^1^Department of Psychiatry and Behavioral Science, Meharry Medical College, Nashville, TN, USA

**Keywords:** Frendak vs. United States, Phenis vs. United States, Breivik case, insanity defense, jurisdictions

## Abstract

The imposition of the insanity defense is a complicated psycho-legal scenario. Globally, definitions of insanity differ from country to country. In a multitude of cases, a determination of insanity at the time of a criminal act means the offender will not be considered responsible for his or her action(s). In many jurisdictions, concerns have been raised that the insanity defense has been used to mitigate punishment, usually after a particularly heinous crime. In this review, the authors use three cases – Frendak, Phenis, and Breivik to demonstrate how the imposition of the insanity defense has been used for legal purposes in the past and present. In an effort to give more background to each of the above-mentioned cases, the writers have provided some details to aid comprehension. The authors offer recommendations for the ethical forensic evaluator unburdened by partisan allegiance and invested in the search for truth. This review article relies on peer-reviewed articles available from PubMed, Meharry Online Library, and legal dictionaries. We also cross-referenced reputable news sources to ensure the validity of the facts we present.

## Introduction

Societies, in the main, believe that criminals should be punished for their crimes. At the same time, societies also advocate that laws should not punish defendants who are mentally ill and incapable of understanding and knowing that their actions were wrong and/or were unable to control their conduct (McNaughton Standard, American Library of Law). In this way, the insanity defense reflects a compromise on the part of society and the law (1).

The legal definition of insanity is “a condition which renders the affected person unfit to enjoy the liberty of action because of the unreliability of his behavior with concomitant danger to himself and others” [Ref. (2), p. 794]. Importantly, insanity is not the same as low intelligence or mental deficiency due to age or injury. The legal proceedings following a defense of insanity require psychiatric/medical input to determine whether the defendant be placed in a penal institution or mental-health facility for treatment. In a criminal case, the defendant may plea “not guilty by reason of insanity.” This plea requires a trial or hearing to determine sanity at the time the crime was committed (3).

The concept of willful intent is essential to the determination of whether or not the offender is guilty. A person found to be “insane” is considered incapable of forming such intent. The standard used for determining a defendant to be not guilty by reason of insanity has changed through the years from adherence to strict guidelines, to more lenient interpretations, and back to an increasingly strict standard (4). In the early twentieth century the insanity defense was better defined which decreased ambiguity in its use (5).

**Figure 1:**
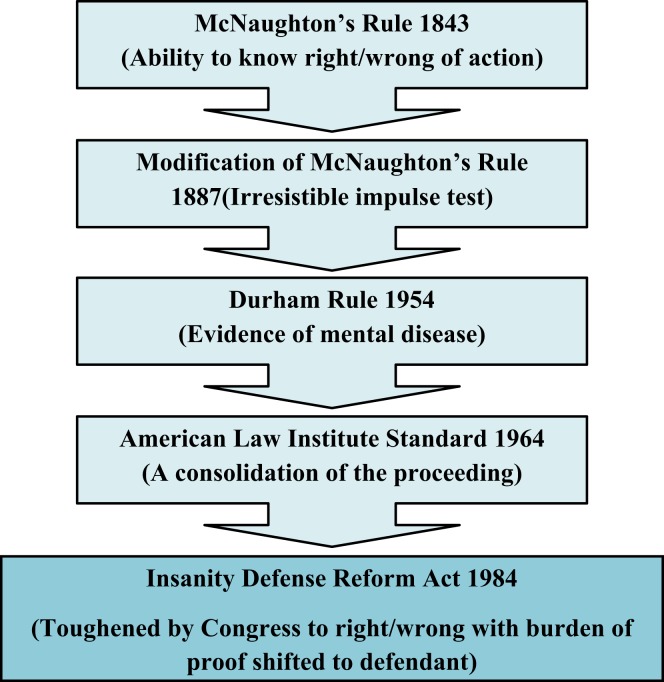
**One evolutionary line of the insanity defense, (GB to US)**.

Figure 1 describes these changes in chronological order (6).

The McNaughton Rule is the basic test for insanity in most jurisdictions in the USA, and emerged as a defense in the US during the nineteenth century (7). In 2009, Bennett demonstrated the inadequacies of the McNaughton Rule (8).

Currently, in the United States, forensic mental-health professionals (psychiatrists, social workers, and psychologists) conduct the determination of whether or not the defendant fits the Black’s Law Dictionary definition of insanity at the time of the crime [Ref. (2), p. 794]. Prior to the above standard definition, forensic evaluators used the “old standard,” a list of test questions designed to determine whether the defendant could distinguish between right and wrong. Large et al. (9) conducted a study to determine the reliability of the expert witness’s evaluation. In this study, the level of agreement regarding not guilty by reason of mental illness (insanity) was moderate to good by expert witnesses of opposite sides (9). Problems remain in cases where the defendant is in discord with his/her attorney(s) regarding the use of insanity as the defense.

In 1979, the precedent of the Frendak inquiry was instituted in response to Frendak vs. United States (10). The Frendak inquiry refers to a process used to determine whether a defendant intelligently and voluntarily waived the insanity defense or not. In Phenis vs. United States (2006), the standard of the Frendak inquiry was revisited. Recently, a new twist on the insanity standard (and a consideration for the imposition of the insanity defense) arose in a high-profile case in Norway. The case in Norway centered on the defense of Anders Breivik, for whom the prosecution and defense have decided to argue the following issues: Did the defendant know right from wrong at the time he carried out the atrocities? Was he suffering from a mental illness? Was he fully capable of separating fantasy from reality? Did he have the ability to conduct his affairs in the absence of psychosis? Was he subject to uncontrollable behavior at the time he committed mass murder?

We use the three cases to illustrate the principle of the Frendak inquiry in the insanity defense (10), the application of the principle in another case in the United States (11), and how it compares to a high-profile international case (12).

The Frendak vs. US (10) case is a landmark case with great educational value for all forensic psychiatrists, especially in North America. It presents an unusual situation where all but the defendant, Paula Frendak, harbored the view that she was insane at the time of the crime. The astute forensic evaluator would be well advised to consider the potential implications of the insanity defense being imposed on the defendant and act accordingly, i.e., after engaging the Frendak inquiry. (The outline has been made available in our manuscript).

In 2006, the Phenis vs. US case went to trial and ended with Mr. Phenis guilty by jury. Several years later, the case was unsuccessfully appealed. The basis for the appeal was the court’s failure to institute the Frendak inquiry. After the application of the Frendak inquiry, the Court of Appeals upheld the guilty verdict in the Phenis case.

Norway’s Breivik case appears in this review to highlight the international nature of attempts to impose the insanity defense. Additionally, it highlights the extremely unusual circumstance where the prosecution was pressing for a verdict of insane while the defense was pressing for a verdict of sane.

## Frendak vs. United States (Frendak vs. United States, 1979)

### Facts of Frendak vs. United States

At approximately 2:15 on the afternoon of January 15, 1974, Mr. Willard Titlow left his office and took the elevator from the seventh floor. Paula Frendak, a co-worker, departed immediately afterwards and within a few minutes Mr. Titlow was found fatally shot on the first floor hallway of their office building.

Following the shooting, Ms. Frendak left Washington, DC, USA, where the incident occurred. She was eventually apprehended on February 11, 1974 in Abu Dhabi for not surrendering her passport at the airport. When searched, she was in possession of a 0.38 caliber pistol, 45 rounds of ammunition, 2 empty cartridges and a pocketknife (13). Authorities in Abu Dhabi surrendered Ms. Frendak to the United States Marshals on March 13, 1974. She was brought back to the District of Columbia and on May 29th of the same year she was charged with 1st degree murder and for possession of an unlicensed pistol.

At the trial, the Government presented evidence that Mr. Titlow had been shot twice. The evidence showed that someone stood over the victim as he lay on the floor and fired the last shot. With the help of a police expert in firearms identification, tests showed positively that the bullets removed from Mr. Titlow’s body had been fired by the weapon seized from Paula Frendak.

Robert Hur, a co-worker, testified that Ms. Frendak had followed him and Mr. Titlow on three (3) occasions prior to January 15, 1974. Another co-worker, Thomas Voit, recalled a similar incident that occurred on the day of the murder. Ms. Frendak had followed him and the deceased as they left the office and were taking the elevator. Mr. Titlow tried to avoid Ms. Frendak telling her that he and Mr. Voit were going out to eat. Both men left for the cafeteria and realized that Ms. Frendak had followed. She took the elevator up with them.

Additionally, a secretary in the office testified that immediately preceding the shooting Ms. Frendak had followed Mr. Titlow as soon as he had left the office for his regular sales call. He was found fatally wounded a few minutes later. Paula Frendak admitted to ownership of the murder weapon and claimed she had brought it to sell to Mr. Titlow. She had left the office with him in order to complete the transaction. After handing the pistol to Mr. Titlow, an unknown woman grabbed the gun from the deceased, shot him twice, and fled. Paula Frendak claimed she panicked and left the city in the aftermath.

In the months preceding her trial, Ms. Frendak underwent four competency evaluations to assess her mental status and her ability to consult with counsel on matters related to the case. After the fourth hearing, the Court found that she was suffering from a personality disorder, but was deemed able to consult with counsel concerning the proceedings against her. The Court concluded that Ms. Frendak was competent to stand trial and subsequently found her guilty of first-degree murder and carrying an unlicensed pistol (13).

### Issue on appeal

**Figure 2:**
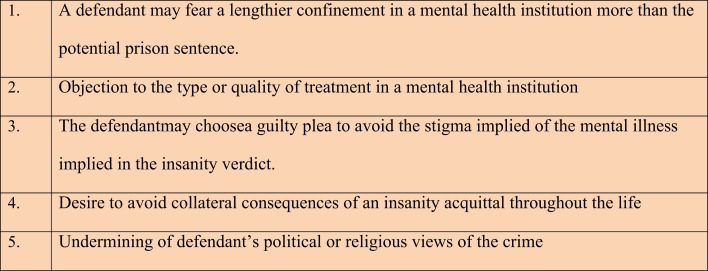
**Defendant’s potential (rational) objections to an insanity defense, (from forensic neuropsychology: a scientific approach, page 456, by Glenn J. Larrabee)**.

Prior to sentencing, the judge ordered a criminal responsibility evaluation to determine her mental state at the time of the offense. The Trial Court overruled the conviction and found her to be “Not Guilty By Reason of Insanity” even though she refused to plead insanity and appealed. Later, the District of Columbia Court of Appeals concluded that a trial judge cannot force an insanity defense on a defendant who is competent to stand trial if the defendant intelligently and voluntarily decided to reject the insanity defense (14, 15). The Court listed five legitimate and rational reasons for which a defendant might reject the insanity defense (Figure 2).

### Holding on appeal

In the Frendak case, the government produced sufficient evidence to support a conviction for first-degree murder. However, due to the challenge created by the second issue, the appeals court ruled that a trial judge might not force an insanity defense on a defendant found competent to stand trial if the individual intelligently and voluntarily decides to forego that defense. The Court of Appeals decided that the lower Court’s finding of “Competency to Stand Trial” was not sufficient to show the defendant capable of rejecting an insanity defense. The higher court also instructed the trial judge to make further inquiry into whether the defendant had made an intelligent and voluntary decision. It was unclear whether Paula Frendak had made such a decision. Therefore, the decision of the court was reversed, and she was remanded for the additional proceedings (16).

### Reasoning on appeal

To avoid the confusion alluded to above in a Frendak-style jurisdiction, it is valuable for the Forensic Examiner to be aware of potential reasons that a defendant may be rejecting the insanity defense. Moreover, it is crucial to assess the impact of any mental illness on the defendant’s ability to make an intelligent and voluntary judgment (A.K.A. willful intent).

In certain circumstances, while the Frendak inquiry allows the courts to raise the insanity defense for a defendant, it has also upheld the societal concept of justice in which the defendant has been found incompetent to waive the defense. For a defendant who is otherwise competent to stand trial, a decision to waive the defense for any of the reasons listed above (at least in a jurisdiction following Frendak) would most probably be respected (17).

The Frendak inquiry is a three part inquiry that includes (1) an inquiry into competency to stand trial, (2) if the defendant is competent to stand trial, then an inquiry into whether or not the defendant has the capacity to voluntarily waive the insanity defense, and (3) whether the court, on its own will, should impose the defense based on evidence of the defendant’s mental condition at the time of the crime. The Frendak inquiry is of considerable value to legal proceedings. It has become a pivotal part of the proceedings in many other cases such as in Phenis vs. United States (11).

## Phenis vs. United States (District of Columbia Court of Appeals – Phenis vs. United States, 2006)

Phenis vs. United States relates to the insanity defense as well as to the question of an imposed insanity defense in Frendak. Jamar Phenis was convicted of arson, malicious destruction of property and second-degree cruelty to children. Phenis appealed, claiming that the court should have ordered a competency evaluation during the pre-trial portion of his case, that the court failed to do a Frendak inquiry, that the court improperly precluded Phenis from defending against the specific element of arson, that there was an error in the arson jury instruction, and that the trial court erred when it corrected the appellant’s illegal sentence (18). The judges (Ruiz, Glickman, and Schwelb) found the claims to have no merit, except for the Frendak inquiry.

### Facts of Phenis vs. United States

In order to understand how the Frendak inquiry pertains to Phenis vs. United States, it is helpful to know the facts of the case and the timeline of events from pre-trial to sentencing.

On June 27th, 2000, maintenance workers were called to investigate a broken window at the apartment complex where Jamar Phenis lived with his mother. When they arrived at the apartment, they found Jamar Phenis arguing with his mother. The workers also noted a broken patio door and a shattered window. Maintenance left the apartment a few minutes later and at that stage, the argument escalated. Shortly afterwards, Jamar Phenis’ mother, Ardis, arrived at the property manager’s office and asked the manager to call the police. Maintenance workers then returned to the apartment and witnessed a chair on fire being thrown off the balcony. They also witnessed Jamar Phenis’ 6-year old niece, Nigeri Cooper, run out of the apartment horrified by her uncle’s behavior. She said that her uncle had “set the place on fire.” The remaining residents were evacuated. The maintenance workers observed Mr. Phenis strolling out of the building. He did not call for help or report the fire. The workers notified the police that Mr. Phenis had started the fire and he was summarily arrested.

During questioning, Jamar Phenis stated, “Well, I guess I did it. I struck a couple of matches … I threw the first match on a pile of newspaper. I threw [the second match] on the couch.” The question of whether or not Mr. Phenis deliberately set the fire or was unsuccessful in putting it out was argued during the trial.

During the pre-trial proceedings conducted by Dr. Lawrence Oliver, a clinical psychologist who conducted a competency examination, Mr. Phenis was found to have “judgment and insight distorted by unrealistic thinking.” Later, a court order issued on July 12, 2000, instructed Dr. Oliver to conduct a complete competency examination at the mental-health unit of the District of Columbia jail.

Dr. Oliver was unable to complete the examination because Mr. Phenis refused to participate. Subsequently, Dr. Oliver found Mr. Phenis to be incompetent to stand trial (IST) due to mental-health concerns. He cited facts such as Mr. Phenis not bathing for several weeks, refusing to take his medications and not attending his appointments at the clinic. Dr. Oliver evidenced his opinion regarding Mr. Phenis’ unrealistic thought processing by revealing the defendant’s current point of view, “I’m ready to return to society. They should give me bond.”

At the end of the probable cause hearing, Mr. Phenis was found to be IST. The court ordered a further evaluation at St. Elizabeth’s Hospital with an updated competency report to be submitted by October 2000. In September 2000, Dr. Mitchell Hugonnet, staff psychologist at St. Elizabeth’s Hospital, found that Mr. Phenis was competent to stand trial. The court held that Mr. Phenis had a good understanding of the charges brought against him.

Again, in October 2000, Mr. Phenis was found competent to stand trial after he was described as being in control of himself, compliant with his medication and not at risk of danger to himself or to others. However, Mr. Phenis remained at St. Elizabeth’s Hospital to ensure that he would remain compliant and competent to stand trial. Before the trial began on June 25, 2001, the defense asked the court to order a “Criminal Responsibility Test” to assess Mr. Phenis’ mental state at the time of the offense.

The defense specifically declined to request or pursue the Not Guilty by Reason of Insanity plea, but wanted to develop information regarding their theory that he had a mental illness at the time of the crime. Mr. Phenis specifically denied a plea of Not Guilty by Reason of Insanity.

In August of 2001, Dr. William Richie, a staff psychiatrist in the Forensic Inpatient Services Division of the District of Columbia Department of Mental Health, concluded after his evaluation of Mr. Phenis, that Mr. Phenis was not suffering from a mental disease or defect that could have caused him to be incapable of recognizing the wrongfulness of his actions. Dr. Richie’s report made it difficult for the defense to pursue a plea separate from Not Guilty By Reason of Insanity. Mr. Phenis’ condition was subject to deterioration and he was required to remain at St. Elizabeth’s to ensure continued competency.

In October 2001, the defense informed the judge that Mr. Phenis wanted to offer a plea of guilty to the charge of malicious destruction of property. This was contingent on the government dismissing the two other charges, waiving enhancement papers, and reserving the right to ask the trial court to hold the appellant in jail pending sentencing.

A District of Columbia Superior Court jury found Jamar Phenis guilty of Arson and Malicious Destruction of Property and Second Degree Cruelty to Children. Phenis was sent back to St. Elizabeth with pending sentencing. Soon afterward, a hearing was conducted on January 29, 2002, to hear the request by St. Elizabeth’s for Mr. Phenis to be transferred to jail. The judge ordered for another mental-health examination for Mr. Phenis, this time conducted by the District of Columbia’s Forensic Services Administration.

On January 31, 2002, Mr. Phenis was transferred from St. Elizabeth’s to the District of Columbia jail’s mental-health ward where he was evaluated by Dr. Janet Fay-Dumaine. She determined that Mr. Phenis’ condition worsened significantly when he was not on medication and that he needed “intensive mental health and substance abuse treatment.”

At Mr. Phenis’ sentencing hearing on March 20, 2002, he stated that he had been “hallucinating and intoxicated at the time of the fire.” He said he was “sick” and that his mother also was not well. Judge Motley recommended that Mr. Phenis be sent to the Federal Corrections Center in Butner, North Carolina to complete a 9- to 27-year sentence.

### Holding on appeal

In the appeal of Phenis vs. United States (2006), the judges found that it was not clear if Phenis was fully informed of the circumstances surrounding the insanity defense or that he freely chose to waive it. Therefore, the court remanded for a Frendak inquiry.

### Reasoning on appeal

In addition to the belief that the Frendak inquiry had merit in the Phenis vs. United States (2006), the judge offered an opinion on the premise of the Frendak inquiry and stated, “Merely because a criminal defendant may lack the capacity to waive an insanity defense does not mean that it is necessarily the judge who should decide whether that defense should be pursued.” The judge opined that there are alternatives, e.g., appointing a guardian to investigate and make the choice for the defendant, but that would be an issue for a later time.

Ultimately, after the Frendak inquiry was conducted, and due to Mr. Phenis’ continued vehement refusal of the insanity defense, his guilty verdict was finally affirmed on June 25th, 2009. He was returned to Allenwood Federal Penitentiary to serve out the term of his sentence.

## Case of Anders Breivik

The recent high-profile case of Mr. Anders Breivik, the Norwegian gunman, poses an interesting perspective to the application (and potential imposition) of the insanity defense. Mr. Breivik, admittedly, killed 77 individuals in bomb and gun attacks on July 2011 in Norway and admitted that he had done it in defense of his country.

On that day, Mr. Breivik drove a van loaded with explosives to Central Oslo. He detonated these devices outside the office of the Prime Minister, killing eight. Mr. Breivik then traveled 45 km away to Lake Northwest of Oslo, arriving there approximately 90 min after his first attack. At the lake, he disguised himself as a police officer and boarded a ferry headed to Utoeya Island. After a 30-min trip, he disembarked and began shooting participants of a Labor Party summer camp. The victims of his savagery included teenagers attending the summer camp. Mr. Breivik would later confess to all charges against him. However, he refused to plead guilty to committing to any crime and instead claimed “self-defense.”

In his defense, Mr. Breivik explained that his actions were in alignment with the views of extreme right wing militants, a growing and disenchanted faction in many European countries. Mr. Breivik told the judges that he acted in defense of his nation and though he conceded that his actions were cruel, he found them necessary. Just before he began his killing spree, he released a manifesto online to his Facebook followers, and a link to a video on You Tube through which he lambasted the “multiculturalists” whom he claimed are aiding the destruction of European society.

It is most interesting that Breivik’s defense is steadfast on the claim that the defendant’s actions were that of a sane man who felt he needed to preserve the “basics of the European Christian cultural legacy.” For followers of the case in the US, the theory of defense in this case is decidedly unusual, as an insanity defense can mean a mitigated sentence. It seems peculiar that a defense lawyer would encourage a client to plead guilty with willful intent when doing so would usually beckon the full wrath of the law. However, the situation becomes clear upon examination of the criminal justice system in Norway.

In Norway, a defendant found mentally ill at the time of a crime, and is currently mentally ill, will be sent to a hospital for treatment. In addition, public safety is considered a priority when the patient is suffering from a mental disease or defect when the crime is committed, but is not currently afflicted (7, 19).

Interestingly, Norway does not have the death penalty (20). Norway’s legal system allows Mr. Breivik to face a maximum sentence of 21 years if declared sane (though this can be increased incrementally after completion of his sentence by the court’s discretion). Conversely, if he is found to be “insane,” he can be sentenced to a mental institution for as long as he is considered sick and dangerous to others. The prosecution for the case has urged the court to consider Breivik insane, presumably, so that he be held for a longer duration (perhaps, for rest of his life). On the other hand, the defense is arguing for the prospect of a determinate sentence brought about by a verdict of guilty (21, 22).

It is clear that Mr. Breivik wants his actions to be taken seriously. Lene Wold of the magazine *The Independent* writes about Breivik as a self-proclaimed political activist, and if he is sent to a mental hospital that would be in Breivik’s own words, “the ultimate humiliation… a fate worse than death.” Mr. Breivik has gone on to opine that, “history shows, you have to commit a small barbarism to prevent a larger barbarism.” With this rationalization of the crime, one could reasonably conjecture that Mr. Breivik is hoping that he has set the proverbial ball rolling down the hill. According to Geir Lippestad, counsel for the defense, Mr. Breivik’s actions were not delusional but a “part of a political view shared by other right wing extremists.” Olivier Truc of the magazine LaMonde quoted Mr. Lippestad’s revelation that, “We will place people from extremist backgrounds on the witness stand to explain their thought process in order to establish that there are others who, without going as far as to commit the crime, share the same ideology and way of thinking.” Lippestad said that “[w]hat we want to show is that we are dealing with an ideology and that he is not the only person to stand behind [those beliefs]; that he is not a psychotic living in a separate world.” At its core, Breivik’s view demonstrates a growing intolerance for what the extremists perceive as the, “Muslim invasion.”

In his 1500 page manifesto, Mr. Breivik expounds, “I don’t support the deportation of non-Muslims from Europe as long as they are fully assimilated (I’m a supporter of many of the Japanese/Taiwan/South Korean policies/principles). However, we should take a break from mass immigration in general (as of 2008 numbers). Any future immigration needs to be strictly controlled and exclusively non-Muslim.” This notion prompts the question on whether or not public sentiment will have any effect on the outcome of this trial. As it appears, the Norwegian public would like to keep Mr. Breivik ensconced in a mental institution where he presumably can be more effectively monitored and restrained.

The use of an insanity defense is controversial (23, 24) and is especially controversial in a high-profile case like Anders Breivik. Approximately 1% of defendants in criminal cases utilize it as a defense, while juries in the United States reject about four of every five insanity pleas (25). We do not have figures available for circumstances where the verdict is the result of “an agreed order” but given the increasing burdens placed upon the criminal justice system (and the propensity for most criminal verdicts to receive a “plea bargain” disposition), we can conjecture that there are many.

This high-profile case has put Norwegian law under the microscope. Dr. Landy Sparr of the Oregon Health and Science University offered some insight into Norway’s legal system as it relates to the insanity defense. In the journal *Live Science*, the journal’s senior editor Stephanie Pappas authored an article entitled, “What ‘Insanity’ Means for Norwegian Gunman.” She quotes Dr. Sparr’s writing, “In Norway, defendants qualify for an insanity defense only if they can prove they were in a state of psychosis and not in control of their own actions during the crime” (25). Additionally, she pointed out that “Some US states have a test for insanity that is similar to the one used in Norway.” Parenthetically, these “similar” state jurisdictions utilize an “irresistible impulse” or “volitional prong.” Also, of note, it would appear that Mr. Breivik’s first mental-health determination (announced on Tuesday, November 29, 2011) was apparently what would be considered to be a competency to stand trial evaluation, in that it was a preliminary proceeding to be followed by a criminal responsibility determination to be made at a later date. Karen Franklyn, in her online commentary titled “In The News,” dated Wednesday April 16, 2012, observed that Mr. Breivik had a pre-trial evaluation, “what we in the US refer to as a competency hearing.”

Forty-six US states have some version of the insanity defense on the books, with Utah, Montana, Idaho, and Kansas abolishing it. This defense was designed to divert people from incarceration who are incapable of understanding or controlling their criminal actions, and to help them get treatment (26). A Frontline article, entitled “From Daniel McNaughton to John Hinckley,” scrutinized the insanity defense in its circuitous trajectory.

Mr. Breivik was assessed twice (11/2011 and 04/2012) by psychiatrists and was given two different diagnoses: paranoid schizophrenia and narcissistic personality disorder. If Mr. Breivik had the more serious diagnosis of paranoid schizophrenia, there has been no information released to the public that verifies or confirms antecedent behavior consistent with the condition.

Furthermore, Mr. Breivik never admitted to being preoccupied with delusions or auditory hallucinations. Mr. Breivik planned his actions meticulously over time. He equipped himself and selected with consideration specific targets to complete his mission: he admitted to making calculations and decisions on whether or not he should attack a school with younger children or attack a Labor Party summer camp instead. For some, based on the information presented, Mr. Breivik appeared to be in control of his actions, as he rationally executed his crusade. As discussed previously, the paranoid-type schizophrenia diagnosis announced by the prosecutor on November 29, 2011 seems to have been a strategic prosecutorial move, especially considering the lack of corroborating history in the defendant.

Through this case, the question arises as to whether or not the monstrosity of the crime automatically categorizes one as mentally ill and, therefore, qualifies for the insanity defense. If it does, then according to this logic, the terrorists who committed the atrocities in Oklahoma City and on 9/11 may have all been insane. This argument may be dismissed as rhetorical for at least two reasons:

1. An insanity defense is rarely successful when the person committing a crime has an accomplice (as was the case in the Oklahoma City bombings and the attacks on New York, Washington, and Shanksville.) [Ref. (27), p. 647].
2. In order to assert that someone is insane, the evidence should at least be consistent with the minimum diagnostic criteria set for that illness. If Mr. Breivik suffered from paranoid schizophrenia, it follows that he would have met the DSM-IV-TR criteria for a diagnosis.

Additionally, Mr. Breivik prefaced in his manifesto that the alacrity to judge him as insane would be an affront to those who are mentally ill. If the legal system should find Mr. Breivik insane, one could interpret this to be an apparent attempt to address or assuage those who would prefer to avoid the stigma that intolerant Norwegians like Breivik exist. On August 24, 2012, the court decided that Anders Breivik was criminally responsible for his behavior. The prosecution has registered its intent to appeal the decision.

The authors implore that all reasonable forensic mental-health professionals have their attention focused on the way that the Norwegian legal system handled this case. It will be interesting to see if the Frendak inquiry makes its way as precedent into the Norwegian court (should the prosecution appeal a verdict of insanity). As in the Frendak and Phenis cases, forensic mental-health professionals assigned to this case ought to consider engaging in a Frendak-like inquiry prior to an official order or risk having blame attributed to them after the fact for not having done so initially.

## Current Considerations

According to Dr. Miller in 2002, “At least 17 jurisdictions permit insanity defenses to be entered over the objections of defendants” (28). In the same document, he advised that “forensic evaluators” consider “the implications of (the)” position (that) “the major reason for permitting such imposed defenses is a policy preference for preserving the dignity of the law.” Forensic evaluators do not have as their major goal the preservation of the dignity of the law. Rather, a forensic evaluator is motivated by the search for the truth. Perhaps evaluators working in these 17 jurisdictions need consider a pre-emptive exploration of the Frendak inquiry with the defendant, whether asked to do so or not. Currently, 4 states (Utah, Montana, Idaho, and Kansas) have disallowed the insanity defense; therefore, forensic evaluators in these states need not be as concerned that they will retroactively be criticized for neglecting to conduct a Frendak inquiry, when they were not asked to do so initially.

## Conclusion

Paula Frendak’s case illustrated a situation where all parties but her concurred with a determination of her insanity. The case outlined circumstances where an insanity defense might be imposed on a competent defendant, setting the precedent for the “Frendak inquiry.” Jurisdictions where Frendak is law have wrestled with this concept ever since.

Jamar Phenis’ case illustrates a situation where an attempt was made to use the “Frendak inquiry” *ex post* facto and on appeal. This resulted in the guilty verdict being upheld, but raises the issue of whether or not evaluators should engage in a Frendak inquiry whether asked to or not.

Anders Breivik’s case illustrates a situation where, in a reverse of the dominant paradigm, the prosecution attempted to obtain a Frendak-like outcome. The prosecution and the defense were not in agreement here. Mr. Breivik’s wishes to avoid the insanity defense imposed upon him held sway and he was found guilty in the trial court (29). The prosecution has registered intent to seek appeal.

The three cases described are similar in the following ways: (1) there were multiple pre-trial competency evaluations, (2) no Frendak inquiry was ordered during the pre-trial period, (3) the defense declined to mount an insanity defense or request an evaluation for insanity, and (4) the crimes committed in each of the cases would be classified as “Class-A” felonies in the United States. In 17 states of the USA, the death penalty is a potential outcome when the jury or judge issues a guilty verdict in some cases of a “Class-A felony.” Outside of those states, a guilty verdict in a “Class-A” felony can result in life in prison. The above cases were tried in jurisdictions without the death penalty.

According to Dr. Miller in 2002, there were 17 jurisdictions in the US where Frendak is law. Coincidently, there are currently seventeen states where there is no death penalty. Further research should be directed toward identifying those jurisdictions where Frendak is law and at the same time, the death penalty is not applied. In addition, efforts should be made to simplify the law in this complex area by implementing a more rational approach (30). Regardless of the co-occurrence of Frendak and life without parole, in the search for truth, the informed evaluator would be well advised to consider engaging the defendant in a Frendak inquiry whether asked to do so or not.

## Conflict of Interest Statement

The authors declare that the research was conducted in the absence of any commercial or financial relationships that could be construed as a potential conflict of interest.
